# Supplementary material for: Resolving the full spectrum of human genome variation using Linked-Reads
Source: Genome Res. 2019 Apr;29(4):635–45. doi: 10.1101/gr.234443.118 (PMC6442396; doi:10.1101/gr.234443.118)
Supplement: Supplemental Material [file supp_gr.234443.118_Supplemental_longranger-2.2.zip › longranger-2.2.tar.bz2!/lr2.2-public/lib/stan/cmdstan/stan/doc/api/html/trigamma_8hpp_source.html]

Stan: src/stan/math/functions/trigamma.hpp Source File


|  |  |
| --- | --- |
|  | Stan  2.5.0  probability, sampling & optimization |


- Main Page
- Related Pages
- Namespaces
- Classes
- Files

- File List
- File Members

All Classes Namespaces Files Functions Variables Typedefs Enumerations Enumerator Friends Macros Pages

- src
- stan
- math
- functions

trigamma.hpp

Go to the documentation of this file.

1 #ifndef STAN\_\_MATH\_\_FUNCTIONS\_\_TRIGAMMA\_HPP

2 #define STAN\_\_MATH\_\_FUNCTIONS\_\_TRIGAMMA\_HPP

3

4  // Reference:

5  // BE Schneider,

6  // Algorithm AS 121:

7  // Trigamma Function,

8  // Applied Statistics,

9  // Volume 27, Number 1, pages 97-99, 1978.

10

11 #include <stan/math/constants.hpp>

12

13 namespace stan {

14

15  namespace math {

16

46  template <typename T>

47  inline

48  T

49  trigamma(T x) {

50

51  using std::floor;

52  using std::sin;

53

54  double small = 0.0001;

55  double large = 5.0;

56  T value;

57  T y;

58  T z;

59

60  //bernoulli numbers

61  double b2 = 1.0 / 6.0;

62  double b4 = -1.0 / 30.0;

63  double b6 = 1.0 / 42.0;

64  double b8 = -1.0 / 30.0;

65

66  //negative integers and zero return postiive infinity

67  //see http://mathworld.wolfram.com/PolygammaFunction.html

68  if ((x <= 0.0) && (floor(x) == x)) {

69  value = positive\_infinity();

70  return value;

71  }

72

73  //negative non-integers: use the reflection formula

74  //see http://mathworld.wolfram.com/PolygammaFunction.html

75  if((x <= 0) && (floor(x) != x)) {

76  value = -trigamma(-x + 1.0) + (pi() / sin(-pi() \* x))

77  \* (pi() / sin(-pi() \* x));

78  return value;

79  }

80

81  //small value approximation if x <= small.

82  if (x <= small)

83  return 1.0 / (x \* x);

84

85  //use recurrence relation until x >= large

86  //see http://mathworld.wolfram.com/PolygammaFunction.html

87  z = x;

88  value = 0.0;

89  while (z < large) {

90  value += 1.0 / (z \* z);

91  z += 1.0;

92  }

93

94  //asymptotic expansion as a Laurent series if x >= large

95  //see http://en.wikipedia.org/wiki/Trigamma\_function

96  y = 1.0 / (z \* z);

97  value += 0.5 \* y + (1.0 + y \* (b2 + y \* (b4 + y \* (b6 + y \* b8)))) / z;

98

99  return value;

100  }

101  }

102 }

103

104 #endif

stan::math::trigamma

T trigamma(T x)

**Definition:** trigamma.hpp:49

stan::agrad::sin

fvar< T > sin(const fvar< T > &x)

**Definition:** sin.hpp:14

stan::agrad::floor

fvar< T > floor(const fvar< T > &x)

**Definition:** floor.hpp:15

stan::math::positive\_infinity

double positive\_infinity()

Return positive infinity.

**Definition:** constants.hpp:114

stan::math::pi

double pi()

Return the value of pi.

**Definition:** constants.hpp:77

constants.hpp


---

[ Stan Home Page ]

*© 2011–2014,
Stan Development Team.*
